# Psychometric properties and validation of the English version Giessen Subjective Complaints List (GBB-8)

**DOI:** 10.1186/s40359-022-00741-8

**Published:** 2022-03-11

**Authors:** Katja Petrowski, Markus Zenger, Bjarne Schmalbach, Christina Diane Bastianon, Bernhard Strauss

**Affiliations:** 1 Department of Internal Medicine III, Dresden University of Technology, Fetscherstrasse 74, 01307 Dresden, Germany; 2 Medical Psychology and Medical Sociology, University Medical Center of the Johannes Gutenberg University of Mainz, Mainz, Germany; 3 Faculty of Applied Human Studies, University of Applied Sciences Magdeburg-Stendal, Stendal, Germany; 4 Integrated Research and Treatment Center Adiposity Diseases - Behavioral Medicine, Psychosomatic Medicine and Psychotherapy, University of Leipzig Medical Center, Leipzig, Germany; 5 Institute of Psychosocial Medicine and Psychotherapy, University Hospital Jena, Jena, Germany

**Keywords:** Giessen subjective complaint list, English version, Psychometric properties, Validation, Assessment, Psychosomatic

## Abstract

**Background:**

The present study investigated the psychometric properties of the newly developed English version of the Giessen Subjective Complaint List-8 (GBB-8), a questionnaire assessing psychosomatic symptoms with regard to exhaustion, gastrointestinal, musculoskeletal and cardiovascular.

**Methods:**

A U.S. sample of 638 participants (47.6% female) was recruited by MTurk to participate in this cross-sectional online survey. Validation instruments included the Patient Health Questionnaire-4, Perceived Stress Scale, short version of the Trier Inventory for Chronic Stress.

**Results:**

Reliability was high with ω’s between .80 and .86 for all subscales. Confirmatory factor analyses yielded comparable good model fit for a four-dimensional model as well as a higher order model. Multi-group confirmatory factor analyses confirmed measurement invariance of the GBB-8 across sex and age. Regarding convergent validity, correlations with other instruments were highly significant and of large magnitude as expected.

**Conclusion:**

The English version of the GBB-8 has shown excellent psychometric properties. Therefore, it can be recommended for the assessment of psychosomatic complaints in contexts where short screening instruments are necessary.

## Background

Somatic complaints are highly represented in society [1–3] as well as in patients involved in the health care system [4, 5]. Although these are not directly connected to medical conditions, patients with severe medical conditions commonly utter somatic symptoms. However, somatic symptoms are associated to symptoms of anxiety and depression [2]. The assessment of somatic symptom stress is key in epidemiological research, since such are known to reduce the health-related quality of life and are related to a greater use of the health care services [2, 6, 7].

So far, the assessment comparability of the somatic symptom burden in the general population has been limited by a lack of agreement on the scales used stated by Zijlema et al. [8]. In their recent systematic review, Zijlema et al. [8] identified 40 self-report somatic symptoms questionnaires and assessed them regarding their usability for large scale population studies. The authors suggested the Patient Health Questionnaire-15 [9] and the somatization scale of Symptom Checklist 90 [10]. However, the Symptom Checklist 90 lacks the brevity needed in epidemiological research. Even for gerontic patients or older patients 15 items of the PHQ might be still too long. Therefore, shorter version of the PHQ (including the PHQ-4) and other scales with the assessment of somatic symptoms should be used. Shorter psychometrically sound questionnaires have several benefits: the drop-out rate as well as the rate of missing values are lower in shorter surveys, and the participants experience less boredom or fatigue.

In German speaking societies, a scientifically sound scale for the assessment of subjective health complaints is the Giessen Subjective Complaints List (Gießener Beschwerdebogen—GBB [11]). The GBB-24 consists of 24 health complaints rated on a Likert scale ranging from 0 (not at all) to 4 (very much). The individual complaints can be aggregated on four scales: exhaustion, gastrointestinal complaints, musculoskeletal complaints, cardiovascular complaints. These scales correspond well to commonly reported symptom clusters [8]. After continuous improvement, the 24-item version of the GBB-24 came into application for the evaluation of physical complaints after medical assessments, social stressors, psychotherapy, symptom strain in minority and marginalized groups. The GBB is also used for basic documentation in psychosomatic medicine and psychotherapy [11]. In order to use the GBB in epidemiological research, a shorter version would be necessary. Therefore, an 8-item brief version of the Giessen Subjective Complaints List was developed. The following criteria were applied for the shortened scale: (1) maintaining the original factor structure and having an equal number of items per factor (as is the case in the original long form), (2) the selected items should be among those with the highest item total-correlation from each subscale, (3) the selected items should have a mean above 0.5 in the general population to avoid floor effects.

Psychometric analyses of the German version of theGBB-8 yielded excellent scale properties with regard to item characteristics and factor structure. The eight symptoms included in the questionnaire are among the top 15 symptoms reported by Zijlema et al. [8] as the most frequently assessed. This shows the relevance of the chosen criteria not only due to their psychometric quality but also regarding their content. A factor structure that allows for the computation of subscales, including norms for each subscale, provides an advantage over measures providing only one overall score. Strong measurement invariance can be largely confirmed regarding gender, age, and age × gender. The factors are more easily interpretable and highlight the specific areas of complaint. Given the norms and the confirmed factor structure, the subscales can be used independently.

In sum, the psychometric properties of the GBB-8 are proven to be excellent. Therefore, to utilize this scale in different languages an English version was translated and the psychometric properties were tested in a native English-speaking population. In order to test the validity chronic stress was assessed since high chronic stress is accompanied with more severe somatic symptoms and lower chronic stress with less somatic symptoms.

## Method

### Participants

The sample is comprised of 638 participants. Males and females were relatively equally represented, 47.6% females and 52.2% males. The majority were aged less than 40 (66%) while 15% were 40–49 years old and 19% older than 49. Most participants reported being married (72.3%) and having a bachelor’s degree education (63%). The participants self-identified as ethnically white (79.9%), black (8.6%), Hispanic (5.2%) and Asian (4.1%). A full overview of sample characteristics is found in Table 1.

**Table 1:** Sociodemographic sample details

	*n*	%
Sex		
Male	333	52.3
Female	304	47.6
Missing	1	
Age group		
Younger than 30	239	37.5
30–39	182	28.5
40–49	96	15
Older than 49	121	19
Marital status		
Single	115	18
Married	461	72.3
Domestic partnership	30	4.7
Separated	21	3.3
Divorced	6	0.9
Widowed	3	0.5
Ethnicity		
Hispanic or Latino	33	5.2
Asian	26	4.1
Black or African American	55	8.6
Native Hawaiian or Other Pacific Islander	2	0.3
White	510	79.9
Multi-ethnic	5	0.8
Preferred not to answer	5	0.8
Education		
High School	50	7.8
Associate’s degree	43	6.7
Bachelor’s degree	402	63
Masters’s degree	121	19
Professional degree	8	1.3
Doctorate degree	8	1.3
Other	4	0.6
Household income		
$0–$1249	168	26.3
$1250–$3499	219	34.3
$3500–$4999	99	15.5
$5000 and more	142	22.3
Preferred not to answer	10	1.6

### Data source

Amazon Mechanical Turk (MTurk) was used to recruit participants living in the United States (U.S.) to complete an online survey, which covered topics of acute and chronic stress and uncertainty, physical complaints, emotion regulation, sleep and health behavior. The survey was implemented using SoSci Survey [12], a German based survey platform.

The survey was posted as a HIT (Human Intelligence Task) on MTurk in Fall 2020. The task asked that workers complete an externally hosted survey in exchange for $0.50. The HIT was titled “20–25 min. Psychological Survey about Stress and Uncertainty” and described as “This survey aims to investigate stress and uncertainty during the COVID pandemic and validate a psychological scale with English speakers”. The HIT was visible only to workers with an acceptance rate greater than 95% and who were residents in the U.S. To prevent workers from completing the HIT twice, a qualification was given to all workers that restricted them from partaking in the second round. After completing the survey, they were given an automatically generated code, which was required to provide in MTurk for payment (no workers were rejected for payment).

Several instructional manipulation checks were embedded in the survey as a response quality check [13, 14]. It asked participants to respond to a separate question using the same response options. If incorrect, they were warned to carefully read the instructions and given a second chance to correctly answer. Further, quality control measures were included such as, 2. response consistency between birthdate and age, 3. open response questions were manually coded in line with the Chmielewski article (14) and 4. time checks on the quickness to complete the questionnaire were all checked. Participants who failed to correctly respond after the warning were excluded from the analysis, which is the most effective method based on the literature (see 14). From the full sample, 28 observations were dropped due to complete missing data and an additional 205 participants were excluded based on the response quality checks. Therefore, the sample was reduced to N = 638. According to one missing in the assessment of sex, the sample size was reduced to N = 637 in cases where the variable sex was used for calculations.

### Measures

The Gießen Subjective Complaints List (GBB-8, [15]) is a short and reliable instrument for evaluating the degree of somatic symptoms. The eight items identify commonly reported complaints whereby participants respond on 5-point Likert. The GBB-8 was translated into English in accordance with the International Test Commission (ITC) Guidelines for Translating and Adapting Tests [16]. The items were translated from German to English by one bilingual expert and then back-translated to German by a second bilingual expert. Comparison and reconciliation of the original and back-translated items was carried out by a group of experts, followed by a second round of forward and back-translation. The English GBB-8 items are included in “Appendix”.

The Patient Health Questionnaire (PHQ-4, [1]) is a 4-item inventory to very briefly identify depression and anxiety. Items stem from the Generalized Anxiety Disorder (GAD-7) and the PHQ-8. Participants rate items on a 4-point Likert scale. The two-factor structure is represented by the two anxiety items (Factor 1) and the two depression items (Factor 2). The two factors explained 84% of the total variation and factor loadings were all ≥ 0.82 [17]. Reliability of PHQ-4 scales are good (Cronbach α > 0.80) [17].

The Perceived Stress Scale (PSS-10) [18] measures the degree to which life has been experienced as unpredictable, uncontrollable and overloaded over the past month. Participants respond on a 5-point Likert scale. Cohen et al. [18] originally developed the PSS as a single factor, however since its development, many researchers have concluded the scale represents two distinct factors: (1) perceived helplessness and (2) perceived self-efficacy [19–21]. The PSS-10 consistently shows strong internal reliability (Cronbach α > 0.70) in diverse populations and meets the criteria for good test–retest validity (> 0.70) [22].

The short-English version of the Trier Inventory for Chronic Stress (TICS-9) is based on the original 57 item scale [23] that was translated into English, shortened and validated [24, 25]. The TICS-9 represents nine factors of chronic stress. These include: Work Overload; Social Overload; Pressure to Perform; Work Discontent; Excessive Demands at Work; Lack of Social Recognition; Social Tensions; Social Isolation; Chronic Worrying. Participants rate the frequency of specific situations over the previous three months on a 5-point Likert scale. The English TICS-9 reflects the strengths of the full 57-item English TICS [24]; as it is reliable (Cronbach α ≥ 0.86), shows good model fit, and the scale structure is invariant between males and females supporting the scale validity [25].

### Statistical analyses

All analyses were conducted in R, using the packages lavaan, moments, multilevel, and semTools [26–29]. There was only a small amount of missing data (166 of 6744 GBB data points; i.e. 2.5%). Nonetheless, we ran confirmatory factor analysis using robust full-information maximum likelihood estimation to deal with missing values and non-normal distributions [30, 31]. For the evaluation of model fit we followed the guidelines provided by Schermelleh-Engel et al. [32]: a non-significant χ^2^, Comparative Fit Index/Tucker-Lewis Index (CFI/TLI) greater than 0.95 (0.97), Root Mean Square Error of Approximation (RMSEA) smaller than 0.08 (0.05), and Standardized Root Mean Square Residual (SRMR) smaller than 0.10 (0.05) for acceptable (good) fit. For CFI, TLI, and RMSEA we used the robust variants [33, 34]. We report McDonald’s ω as a reliability metric [35].

Next, we tested for measurement invariance by comparing CFI and RMSEA between models that did (did not) constrain the measurement parameters (loadings, intercepts, residuals) to be equal between the groups of interest [36]. Specifically, these include the configural (unconstrained), metric (loadings constrained), scalar (loadings and intercepts constrained), and the strict (loadings, intercepts, and residuals constrained) model. ΔCFI and ΔRMSEA should be smaller than 0.010 and 0.015, respectively. Since we incorporated a higher-order construct in our model, we followed the guidelines provided by Chen et al. [37] and tested first- and second-order invariance successively. After establishing strict invariance on both the first and second factor order, we then also examined the latent mean differences by additionally constraining the higher-order factor to be equivalent between groups. In addition to the χ^2^-test, we examined the standardized factor mean difference using the formula:

1 $$f = \frac{{\sqrt {\frac{{\mathop \sum \nolimits_{i}^{k} \left( {{\upalpha } - {\overline{\alpha }})^{2} *n_{i} } \right)}}{{n_{total} }}} }}{{{\uppsi }_{P} }},$$

with

2 $${\overline{\alpha }} = \frac{{\mathop \sum \nolimits_{i}^{k} ({\upalpha }_{i} *n_{i} )}}{{n_{total} }},$$

where ${\uppsi }_{P}$ is the standard deviation of the respective factor, pooled across all tested groups, $n_{k}$ is the sample size of group *k,* and ${\upalpha }_{k}$ is the latent variable mean in group *k*.

## Results

### Item descriptive statistics

In Table 2, we report descriptive item statistics. Skewness and kurtosis for all eight items indicate normal distribution [38]. In addition, we calculated the squared Mahalanobis distance and tested it for significance to identify outlier cases. A total of 4 (0.6%) of cases were flagged as outliers but were retained in the analysis. Removing these cases from the analyses did not meaningfully change the outcomes. The subscale-specific correlations as well as the item-total correlations for the total score were high. This was to be expected as the GBB-8 is a homogenous instrument that assesses a relatively narrow construct.

**Table 2:** Descriptive item statistics

	*M*	SD	Skewness	Kurtosis	*r*_subscale_	*r*_*it*_
Being easily exhausted^Ex^	1.73	1.36	0.06	1.71	.66	.80
Tiredness^Ex^	2.06	1.25	− 0.19	2.01	.66	.74
Feeling bloated or distended^Gast^	1.71	1.36	0.10	1.76	.75	.81
Stomachache^Gast^	1.85	1.38	0	1.72	.75	.81
Backache ^Musc^	1.98	1.30	− 0.12	1.90	.68	.73
Neck or shoulder pain^Musc^	1.95	1.31	− 0.09	1.88	.68	.73
Palpitations or heart pounding^Card^	1.53	1.28	0.21	1.83	.68	.76
Dizziness^Card^	1.7	1.34	0.09	1.74	.68	.79

*M*, mean; SD, standard deviation; r_subscale_, subscale-specific correlations; r_it_, item-total correlations. Subscales: ^Ex^, exhaustion; ^Gast^, gastrointestinal; ^Musc^, musculoskeletal; ^Card^, cardiovascular

### Factorial validity

We tested a total of three different model configurations. First, we tested a unifactorial model, which evinced acceptable fit, χ^2^(20) = 133.636, *p* < 0.001, CFI = 0.965, TLI = 0.951, RMSEA = 0.100, SRMR = 0.030. Model fit improved substantially by grouping the items onto their specific subscales in a four-dimensional model, indicating that the higher-order factor aligns well with the empirical data structure, χ^2^(14) = 57.740, *p* < 0.001, CFI = 0.988, TLI = 0.975, RMSEA = 0.071, SRMR = 0.018. Finally, we expanded the four-dimensional model by adding a second-order latent variable, representing general somatic symptom burden. This model had virtually the same fit as the four-dimensional one, showing that a general construct underlying the four subscales can be assumed, χ^2^(16) = 64.529, *p* < 0.001, CFI = 0.985, TLI = 0.974, RMSEA = 0.072, SRMR = 0.021. In terms of reliability, all subscales evinced good coefficients in the four-dimensional model with a second-order construct, with ω’s between 0.80 and 0.86. In addition, the reliability of the second order factor was excellent by all accounts: The vast majority of variance in both Level 1 (ω_L1_ = 0.922) and Level 2 (ω_L1_ = 0.977) is explained by the second order factor.

### Measurement invariance

We then tested the second-order factor model for measurement invariance across sex and age. As can be seen in Table 3, model fit decreases were negligible upon introducing the various constraints. Thus, the model can be assumed invariant across sex and age. As a result, comparisons of both, latent and observed means and variances, are admissible. Finally, we constrained the latent means of the second-order factor to be equal between groups to check whether there are significant differences between groups. Here it became clear that for sex there was virtually no difference (*p*(χ^2^) = 0.169, *d* = 0.10), whereas for age there were significant but small differences (*p*(χ^2^) = 0.012, *R*^2^ = 0.014).

**Table 3:** Test of measurement invariance across sex and age

	χ^2^	*df*	Δχ^2^	Δ*df*	*p*	*CFI*	Δ*CFI*	*RMSEA*	Δ*RMSEA*
Participant sex (male, female)									
Configural model	81.981	32				.985		.072	
1st order weak invariance	89.422	36	7.441	4	.114	.985	0	.069	.003
2nd order weak invariance	93.273	39	3.851	3	.278	.985	0	.066	.003
1st order strong invariance	99.694	43	6.421	4	.17	.985	0	.063	.003
2nd order strong invariance	107.529	46	7.835	3	.05	.984	.001	.063	0
1st order strict invariance	117.238	54	9.709	8	.286	.984	0	.059	.004
2nd order strict invariance	126.662	58	9.424	4	.051	.982	.002	.06	− .001
Mean equivalence	128.558	59	1.896	1	.169	.982	0	.06	0
Age groups (< 30, 30–39, 40–49, > 49)									
Configural model	108.741	64				.987		.068	
1st order weak invariance	124.371	76	15.63	12	.209	.987	0	.062	.006
2nd order weak invariance	135.123	85	10.752	9	.293	.987	0	.059	.003
1st order strong invariance	145.451	97	10.328	12	.587	.988	− .001	.053	.006
2nd order strong invariance	153.651	106	8.2	9	.514	.988	0	.05	.003
1st order strict invariance	194.985	130	41.334	24	.015	.984	.004	.053	− .003
2nd order strict invariance	202.323	142	7.338	12	.834	.984	0	.05	.003
Mean equivalence	213.344	145	11.021	3	.012	.982	.002	.052	− .002

χ^2^, chi square; *df*, degrees of freedom; Δ, change from previous model; *p*, significance level; CFI, Comparative Fit Index; RMSEA, root mean square error of approximation

### Convergent validity

In Table 4 we report correlations between the GBB subscales and total score and related scales of psychological symptoms. As expected, all correlations were highly significant and of large magnitude.

**Table 4:** Correlation matrix

	1	2	3	4	5	6	7	8	9
1. GBB-exhaustion	–	.79	.73	.77	.91	.65	.66	.75	.65
2. GBB-gastrointestinal		–	.71	.81	.92	.61	.61	.73	.63
3. GBB-musculoskeletal			–	.69	.87	.61	.61	.66	.6
4. GBB-cardiovascular				–	.91	.59	.59	.72	.63
5. GBB-total					–	.68	.69	.79	.7
6. PHQ-anxiety						–	.71	.75	.68
7. PHQ-depression							-	.73	.62
8. TICS								–	.81
9. PSS									–

### Normative Scores

Normative percentile ranks for the GBB subscale and total scores are reported in Table 5 and in Table 6.

**Table 5:** Normative percentile ranks for the GBB subscale scores

Sum score	Exhaustion	Gastrointestinal	Musculoskeletal	Cardiovascular
0	14	20	14	23
1	23	29	21	31
2	32	37	29	40
3	44	44	40	48
4	54	59	53	67
5	73	73	72	77
6	84	87	81	94
7	98	94	97	98
8	100	100	100	100

**Table 6:** Normative percentile ranks for the GBB total scores

Sum score	Percentile rank	Sum score	Percentile rank	Sum score	Percentile rank
0	8	11	36	22	79
1	11	12	38	23	83
2	14	13	40	24	87
3	18	14	44	25	90
4	21	15	46	26	92
5	23	16	52	27	94
6	25	17	55	28	98
7	28	18	62	29	100
8	31	19	66	30	100
9	32	20	72	31	100
10	34	21	76	32	100

## Discussion

The assessment of somatic symptom stress is of high relevance in the context of epidemiological research [2, 6, 7]. In a recent systematic review [8] the Patient Health Questionnaire-15 [9] was primarily recommended as a measure of somatic symptom stress. Since epidemiological studies need short forms of measures, shorter psychometrically sound questionnaires might have several benefits. The short versions of the PHQ, e.g., PHQ-4, do not include items to assess somatic symptom burden. Therefore, the German GBB-8, established in German speaking countries, was translated into English and its psychometric properties were assessed.

The present study showed that the four specific subscales fit well with the four-dimensional empirical model which indicates a good factorial fit of the GBB-8. In addition, the higher-order factor representing general somatic symptom burden aligns well with the empirical data structure as well. It is impressive that this higher-order model had virtually the same fit as the four-dimensional one. Therefore, it can be assumed that a general construct underlies the four subscales. Thus, the model can be assumed invariant across sex and age. As a result, comparisons of both, latent and observed means and variances, are admissible. Finally, we constrained the latent means of the second-order factor to be equal between groups to check whether there are significant differences between groups. Here it became clear that for sex there was virtually no mean difference, whereas for age there were significant but very small differences between the models which can be neglected. Age has a well-known effect on somatic burden. In this respect it is noteworthy that the majority of age of the sample is younger than 40 (66%) and only 19% older than 49. The older age with the higher somatic symptom burden is not present in the sample which limits the generalizability of the age invariance. The psychometric properties of the GBB have to be reevaluated in a larger representative sample with better distribution of age. Furthermore, sampling with MTurk lead to a larger percentage of highly educated (63%) and more married participants (72.3%). The U.S. MTurk population is known to be younger and more educated in comparison to U.S. representative samples [39, 40], this divergence is common among internet users in general and other online survey methods [40]. However, in line with the younger age, MTurk users are less likely to be married [40], which is inconsistent with the current sample. This may be a result of when the samples were collected (e.g. 2016 and 2020), however the U.S. Census Bureau reports a decline in marriage rates from 2009 to 2019 [41]. Marital status and education influence the somatic symptom burden (Petrowski et al. 2015) and might therefore reduce the generalizability of the current results.

Concerning reliability, all subscales showed good reliability even for the second-order construct. The reliability of the second order factor was excellent. Concerning the validity correlations between the GBB subscales and related scales of psychological symptoms were highly significant and of large magnitude. Therefore, a convergent validity is given and proves the excellent psychometric properties of this short instrument.

### Strengths and limitations

The strength of the English version of the GBB-8 is the excellent psychometric properties with the good factorial structure, the convergent validity and the briefness of the inventory showing its usefulness especially for epidemiological surveys. The study also has a few limitations: First, the data was based on a MTurk sampling, which conveys some limitations in generalizability. The MTurk population is not representative of the U.S. population, however studies have shown MTurk samples are comparable to other traditional subject pools [42]. Second, while Instruction Manipulation Checks (IMC) are shown to reduce sample noise due to non-diligent participants (including bots and farmers) [13], by dropping participants who failed the IMC, there is the potential to harm the external validity of the study [13].

## Conclusion

The GBB-8 is a carefully designed instrument that possesses good psychometric properties. In addition, the applicability of the GBB-8 in different subpopulations is a unique characteristic of this instrument. Now it is even applicable in English speaking surveys.

## Data Availability

The datasets used and/or analyzed during the current study are available from Prof. Katja Petrowski: kpetrows@uni-mainz.de on reasonable request.

## Appendix

### English GBB-8 Items

Please consider for a moment which of the symptoms below you suffer from. Please rate the severity of each symptom by checking the box in the corresponding column. If you do not suffer from a symptom please check "not at all". I suffer from the following:

**Table:**

1. being easily exhausted
2. feeling bloated or distended
3. backache
4. palpitations or heart pounding
5. tiredness
6. stomachache
7. neck or shoulder pain
8. dizziness

Response options are rated as 0 “not at all”, 1 “slightly”, 2 “somewhat”, 3 “considerably”, and 4 “very much”.

## Abbreviations

GBB: Giessen Subjective Complaints List [Gießener Beschwerdebogen]
MTurk: Amazon Mechanical Turk
U.S.: United States
HIT: Human intelligence task
ITC: International Test Commission
PHQ: Patient Health Questionnaire
GAD: Generalized anxiety disorder
PSS: Perceived Stress Scale
TICS: Trier inventory of chronic stress
CFI: Comparative Fit Index
TLI: Tucker Lewis Index
RMSEA: Root mean square error of approximation
SRMR: Standardized root mean square residual
IMC: Instructional manipulation check
